# Design and characterisation of frequency selective conductive materials for electromagnetic fields control

**DOI:** 10.1038/s41598-020-76447-x

**Published:** 2020-11-09

**Authors:** I. V. Konoplev, D. W. Posthuma De Boer, C. M. Warsop, M. John

**Affiliations:** 1 Department of Physics, University of Oxford, Keble Road, Oxford, OX1 3RH UK; 2 ISIS, STFC RAL, Didcot, OX11 0QX UK

**Keywords:** Materials for devices, Theory and computation, Structural materials, Composites, Applied physics, Electronics, photonics and device physics, Characterization and analytical techniques

## Abstract

To prevent the electromagnetic (EM) wakefields excitation, protect detectors from damage at a range of installations and facilities including particle accelerators the EM field control is required. Conductive foils or wires providing EM protection and required thermal and mechanical properties are normally used. We suggest novel composite materials with uniquely designed frequency selective conductivity enabling them to overcome the properties of the conventional materials, protect from EM fields and supress undesirable phenomena. Theoretical and experimental investigations are carried out and the conductivity of designed and composite (dual-layer) aluminium/graphene metamaterials as well as graphene and aluminium foils is studied. The EM properties of these materials are compared, and conditions of full and partial electromagnetic transparency are discussed. Results observed allow engineering materials capable of EM field control, instability suppression including those observed in high-intensity particle accelerators and enabling control of an EM field generating media including relativistic charge particle beams.

## Introduction

Research to design new materials with specific properties and functionalities, and studies of their applications in science and industry are exponentially growing activities in physics and engineering^1–29^. Different areas of physics from electromagnetics and acoustics to quantum information and medical physics are considering new, naturally occurring (graphene, fullerenes, perovskites) and engineered (metamaterials, periodic structures) materials to resolve a wide range of challenges and newly discovered materials are already having a strong impact on science and society^1–32^. The low density and relatively high electrical conductivity of graphene^14–16^ makes it possible for this material to be considered as an alternative to aluminium in many applications, while synthetic materials such as metamaterials can offer unique electromagnetic (EM) and mechanical properties which has revived interest in their potential applications. The horizons for both graphene and metamaterials are still being explored^1–32^.

**Figure 1:**
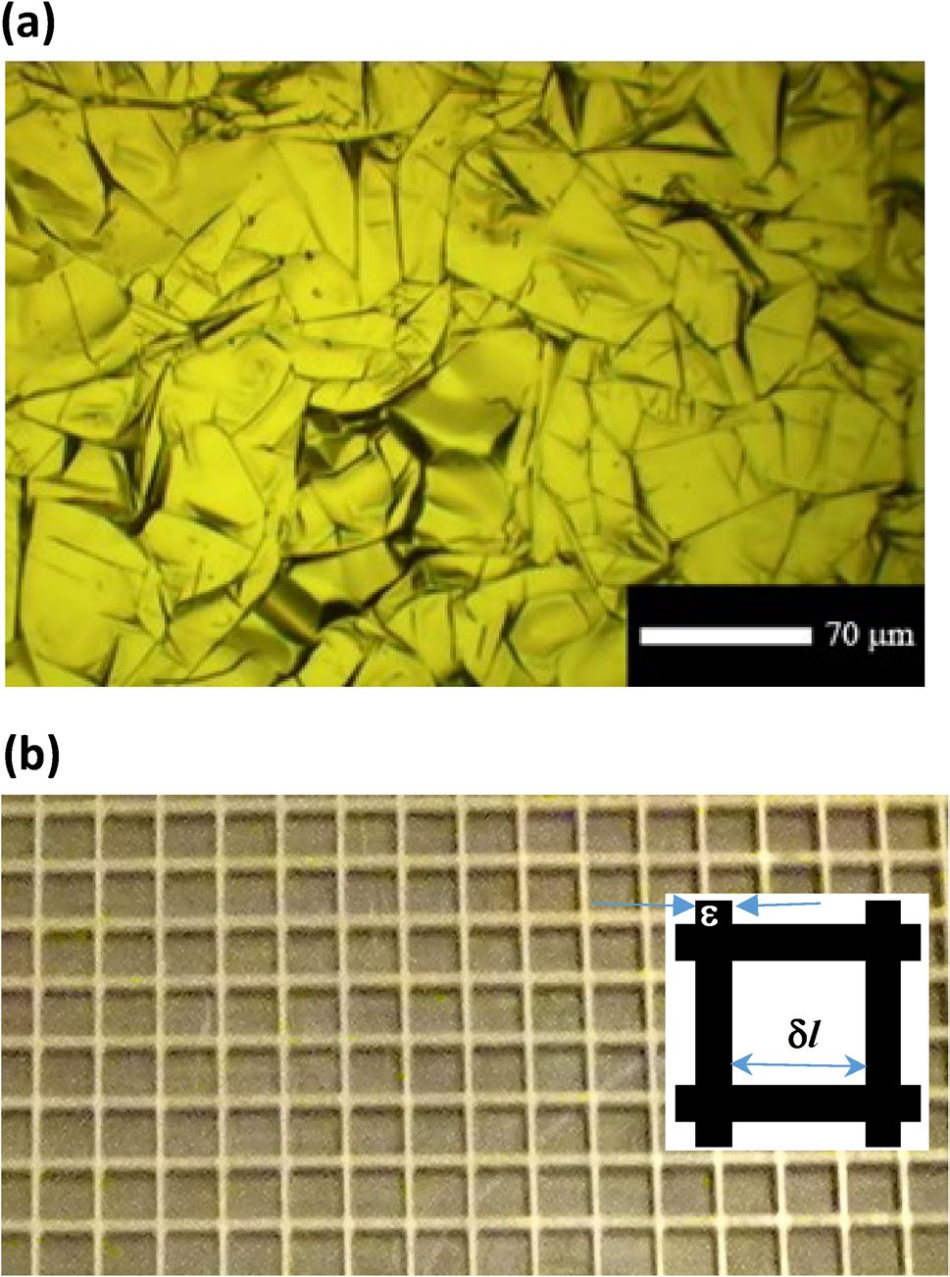
(**a**) Magnified image of the graphene surface used in the experiments; (**b**) photograph of one upper square mesh of thickness 0.4 mm, width of the copper and the dimensions of the square cut. Samples had the same thickness widths of the copper and with the square cuts 2 mm, 4 mm and 6 mm.

Conductive materials are extensively used for shielding detectors from EM radiation in many areas including large research accelerators, light source facilities and detection and communication systems. Detectors are often located in environments with intense EM fields; for example inserted into an accelerator beam line in the vicinity of the charged particle beam^33^. Left unshielded, such insertions lead to the excitation of EM wakefields, which can adversely affect the beam lifetime and detector performance^33^. To prevent EM interference a common practice is to use thin conductive foils to shield the equipment. Here we suggest the concept of materials with frequency selective conductivity and investigate the electromagnetic (EM) shielding properties (i.e. full, partial transparency, reflection) of conductive, single layer foils, novel composite foils (two or more layers) and engineered materials for a range of applications including shielding of VELO (*Ve*rtex *Lo*cater) detector at CERN and prevention of beam instabilities in high intensity charged particle beam accelerators. Figure 1a shows photographs of the graphene sheet structure (multi-domain, polycrystalline available on market) and Fig. 1b shows the metamaterial samples under the investigations. The conductive properties of samples including: aluminium and graphene foils, engineered and composite materials such as aluminium + graphene, aluminium + stainless-steel, copper mesh + conductor and copper mesh + insulator have been studied.

Using the results observed we suggest and demonstrate a way to design materials with a frequency selective conductivity (FSC) allowing EM radiation to be absorbed at one frequency interval while acting as a good conductor at another. The dependence of the materials’ conductivity on the frequency of the EM radiation is studied and discussed. We also discuss the minimisation of the density and thickness of the foil while maintaining its EM properties. The suggested engineered materials have reduced effective density and thickness compared to conventional conductive foils which can be beneficial in many applications. The result presented in this work will benefit many areas including radar, communications, accelerators and high energy physics.

## Findings

### Challenges, observations and results

**Figure 2:**
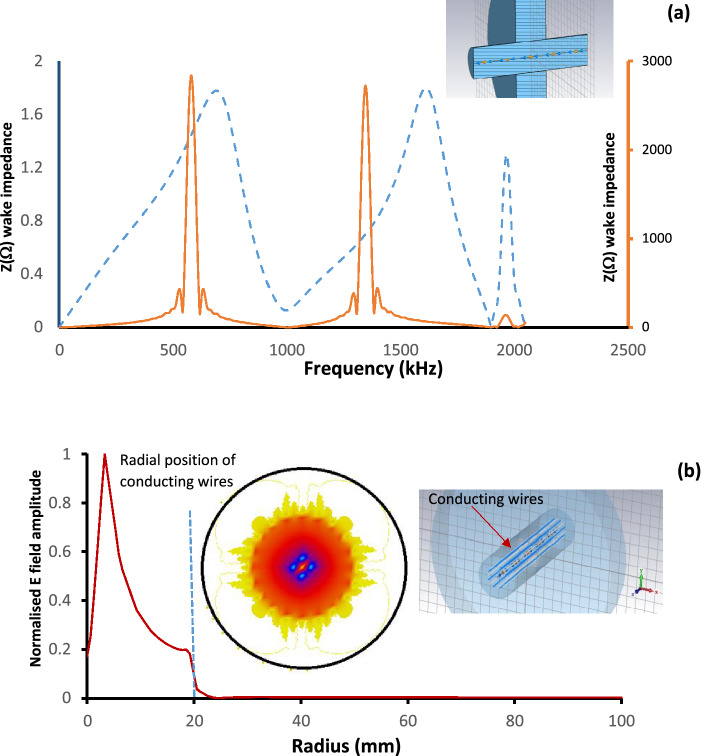
(**a**) The beam coupling impedances of a pillbox cavity that is unshielded (solid line) and shielded by a wire cage (dashed line). The inset illustrates the set-up used in CST particle studio to study the impedances (the electron beam propagates in the middle of the system). (**b**) Dependence of the normalised electric field amplitude on the radius if the beam is shielded. The beam propagates at *R* = 0 mm, the wires are located at *R* = 20 mm. The inset illustrates the contour plot of the electric field of the shielded beam.

During the design of detectors such as those used at accelerator facilities, one task is to minimise the impact of unwanted environmental effects on the detector, including the design of shielding for different types of interference. Here we will look at the design of materials for the electromagnetic (EM) shielding of detectors located in harsh EM radiation environments. An example of such a detector is the VELO particle detector^30–32^ which is located at the LHC facility (CERN). The sensitive parts of this detector come within millimetres of the colliding relativistic particle beams and to operate it must be shielded from the electromagnetic fields. The inset to Fig. 2a shows an example of a cylindrical cavity typically used to host a detector (the model generated by CST Particle Studio) and it is used as a vacuum chamber to host the VELO detector. In this case the beams are propagating along the axis of the vessel leading to the excitation of resonant EM wakefields.

Conventionally the efficiency of the excitation is characterised (Fig. 2a) by the beam coupling impedance^30–33^; the larger is the impedance the higher the efficiency of the wakefield excitation and stronger is the EM field. In Fig. 2a the beam impedances (simulated) are shown before and after adding the shielding cage (Fig. 2b, inset). The cage (set of wires, Fig. 2b) shields the structure’s discontinuity exposed to the beam preventing the excitation of wakefields and their interferences, improving the beam stability and detector performance. The right hand-side inset (the 3D drawing) to Fig. 2b shows the cavity shielded by a wire-cage. The left hand-side inset illustrates the electric field contour plot showing dependence of the amplitude of the EM field induced by the beam on the radial distance which is demonstrated in details in Fig. 2b. The graphs shows the field dependence on the position with respect to the cage (the radial dependence) and the position of the wires indicated by the broken line. The results obtained using CST LF and Particle Studios^34^ and show that the field amplitude drops rapidly outside the cage.

One of the examples of necessity of the novel engineered materials and complexity of the challenges is VELO (CERN) detector. In the specific case of the VELO detector, which studies the trajectories of collision products, shielding with a wire cage could be ideal as it reduces the beam impedance (Fig. 2a) without interfering with the collision products. However, it has crucial disadvantages which illustrate the complexity of the challenge to shield the detector appropriately: high frequency EM radiation leakage through the wires; EM field enhancement on the wires surface leading to RF breakdown and secondary election emission; the accelerator and detector vacuum systems would need to be shared. In VELO, one of the fundamental RF frequencies, due to the local RF system, interfering with the detectors is around 40 MHz corresponding to a 13 μm skin-layer (for the aluminium). In addition to providing effective electromagnetic shielding, optimal foil thickness also requires consideration of rigidity, total mass and (for VELO) minimisation of the secondary scattering of the collision products. The current VELO detector uses a 250 µm thick foil which provides effective EM shielding and rigidity. Unfortunately it also leads to significant unwanted scattering of the collision products and to improve the detector’s performance, optimised shielding is required, which has to be thinner, have a lower density to reduce scattering; be a good conductor; rigid and not degrading in case of bombardment of relativistic particles (similar to a metal). These requirements are creating a need for an engineered material and in this work we discuss properties of the designed material and engineer frequency selective material to address the challenges.

Aluminium foils are a good alternative to the wire cage. Aluminium is easy to manufacture, has excellent electrical conductivity (3.4–3.77)10^7^ (S/m), relatively low density 2.7 g/cm^3^ and low atomic number 13. The last two parameters are important indicators of probabilities to scatter the collisions’ products and activation of the materials by high energy particles. The foil rigidity and manufacturing availability also lead to constraints on the foils thickness, which can be chosen using the skin depth δ^35,36^, and which arises from the complex dielectric permittivity $\varepsilon = \varepsilon^{\prime} + i\varepsilon^{\prime\prime}$ of a conductive material, resulting in the appearance of the surface impedance:

1 $$ \delta = \sqrt {\frac{\lambda }{\pi c\mu \sigma }} ,\;\;\;\; Z_{surf} = c\frac{1 + i}{\lambda }\pi \mu \delta $$

where c is the speed of light, *i* is the imaginary unit, $\sigma$ is the conductivity,$\lambda$ is the wavelength in vacuum and $\mu$ is the permeability. The expression (1) is derived from the assumptions of the semi-infinite materials and it is normally considered that there is no EM field after the skin layer. One notes that in most cases such an approximation works well as the amplitude of the EM field decays exponentially inside the metal and for the copper $\sigma ={5.96\cdot 10}^{7}$(S/m) the skin layer is 4 μm if the radiation wavelength is 1 m (300 MHz) whilst for aluminium this is 5.2 μm due to the lower conductivity. Expression (1) becomes simplistic in case of multi-layer structures with finite thickness, comparable with skin depths of the materials.

**Figure 3:**
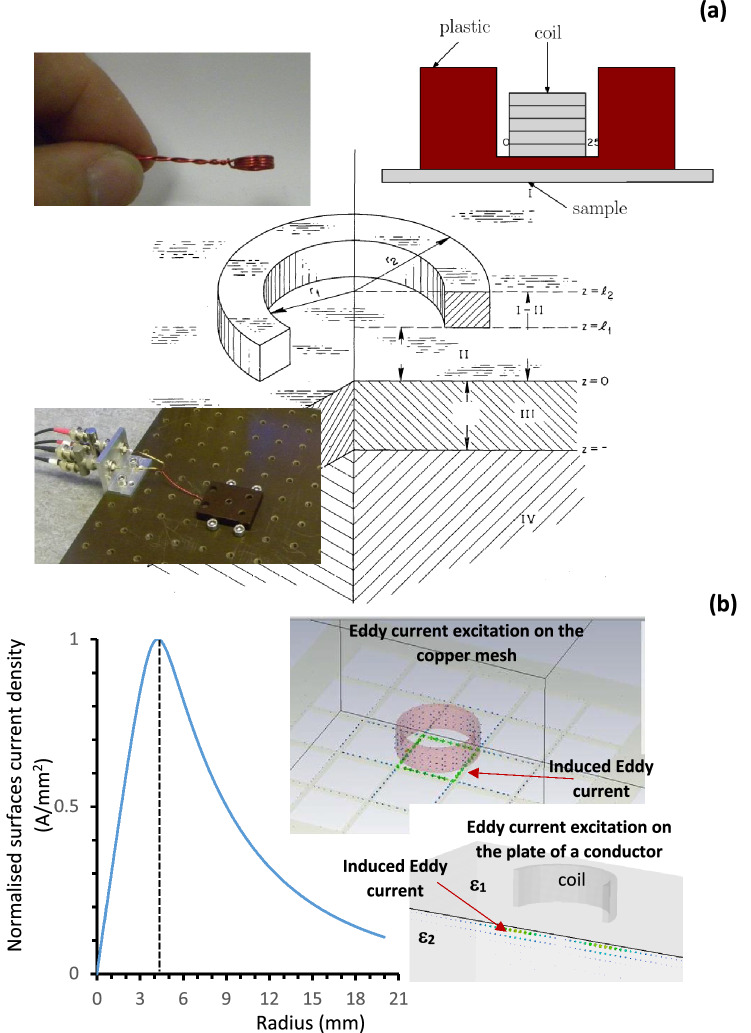
(**a**) Schematic of the experimental set-up. The left top and bottom insets are the photographs of the coil and set-up (respectively) to measure the surface impedance using an Eddy Current technique. The top right inset is the schematic of experimental set up shown in bottom right inset. (**b**) The dependence of the surface current amplitude induced by the time variable magnetic field on the surface of a conductor versus radius and generated by the ideal coil of radius 4.1 mm. The decay of the surface current allowed relatively small samples to be used (40 mm x 40 mm). The inset shows the excitation of surface currents (green arrows) on metal mesh and solid sample. The size of the arrow indicates the amplitude of the current.

One of the experimental methods of evaluating the appropriate foil thickness (in frequency range from Hz up to 1 GHz) for EM shielding is the “Eddy current” technique (Fig. 3a)^37–43^, where Eddy currents are induced in the foils by an excitation coil. In Fig. 3a an illustration of the “Eddy current” model and the geometry of the experiments are shown^37^. This approach is more accurate for studying material properties in the RF frequency range (i.e. from Hz up to 10′s of MHz and as high as 1 GHz if suitably small coils could be constructed) as compared with the direct observation of the transmitted and reflected signals. The distribution of the coil-induced currents on the surface of the samples are shown in Fig. 3b. The insets illustrate the surface current distributions on mesh and bulk materials (top and bottom inset respectively) with the green arrows indicating the current (the size of the arrows shows the current amplitude).

One notes that the coil has a free space impedance *Z*_*ref*_ which has a real part resulting from the finite conductivity of the coil. If a conductive material is positioned in-front of the coil, the induced Eddy currents will change the coil impedance, yielding ${Z}_{ref}+\Delta Z (\omega )$. Experimentally the real part of $\Delta Z(\omega )$ is obtained by subtracting the measured free-space impedance from the impedance with a conducting sample. Analytically, we calculate *Z*_*tot*_(*ω*) of a line current above a conducting surface, which will include $\Delta Z (\omega )$ and the imaginary part of *Z*_ref_. The real part of the line current’s free-space impedance is zero so the *Z*_*tot*_(*ω*) has only real part of $\Delta Z (\omega )$. The general form for *Z*_*tot*_(*ω*) can be obtained from the integral^37^:

2 $$ Z_{tot} \left( \omega \right) = \frac{{j\omega \pi \mu_{0} n^{2} }}{{\left( {l_{2} - l_{1} } \right)^{2} \left( {r_{2} - r_{1} } \right)^{2} }}\mathop \int \limits_{\alpha = 0}^{\infty } \frac{1}{{\alpha^{5} }}I\left( {\tilde{r}_{2} ,\tilde{r}_{1} } \right)^{2} \left\{ {2\left( {l_{2} - l_{1} } \right) + \frac{1}{\alpha }\left[ {2e^{{ - \alpha \left( {l_{2} - l_{1} } \right)}} - 2 + \left( {e^{{ - 2\alpha l_{2} }} + e^{{ - 2\alpha l_{1} }} - 2e^{{ - \alpha \left( {l_{2} + l_{1} } \right)}} } \right)\left( {\frac{{\left( {\alpha + \alpha_{1} } \right)\left( {\alpha_{1} - \alpha_{2} } \right) + \left( {\alpha - \alpha_{1} } \right)\left( {\alpha_{2} + \alpha_{1} } \right)e^{{2\alpha_{1} l_{3} }} }}{{\left( {\alpha - \alpha_{1} } \right)\left( {\alpha_{1} - \alpha_{2} } \right) + \left( {\alpha + \alpha_{1} } \right)\left( {\alpha_{2} + \alpha_{1} } \right)e^{{2\alpha_{1} l_{3} }} }}} \right)} \right]} \right\}d\alpha $$

where $\mu_{0}$ is the permeability of the free space, $r$ is the radial coordinate, $z$ is the axial coordinate, $\alpha_{i} = \sqrt {\alpha^{2} + j\omega \mu \sigma_{i} }$, $\sigma_{1}$ is the conductivity in the region (III), $\sigma_{2}$ is the conductivity in the region (IV), $I\left( {r_{2} ,r_{1} } \right)$ is the dimensionless function $I\left( {\tilde{r}_{2} ,\tilde{r}_{1} } \right) = \mathop \smallint \limits_{{\tilde{r}_{0} = \tilde{r}_{1} }}^{{\tilde{r}_{2} }} \tilde{r}_{0} J_{1} \left( {\tilde{r}_{0} } \right)d\tilde{r}_{0}$, $\tilde{r} = \alpha r$, $J_{1}$ is the first kind Bessel function of the first order and $\alpha \left[ {{\text{m}}^{ - 1} } \right]{ }$ is a separation constant (variable parameter^37^) and *n* is the number of coil windings. For the purpose and goals of the studies the real part of the impedance is important and, omitting the possible deviations of the imaginary parts, all impedances presented are understood to be the real parts of the impedance and we will use ${\text{Re}} \left( {\Delta Z\left( \omega \right)} \right) = {\text{Re}} (Z_{tot} \left( \omega \right)) = \Delta Z\left( \omega \right)$ . In (2) the integration over $\alpha$ arises from a Fourier decomposition of the surface currents excited and $\alpha$ can be considered as the corresponding spatial wave vector. Expression (2) allows to evaluate and describe the dependence of $\Delta Z$ on the incident EM signal frequency $\left( {\Delta ZF} \right)$ for two layers of material.

**Figure 4:**
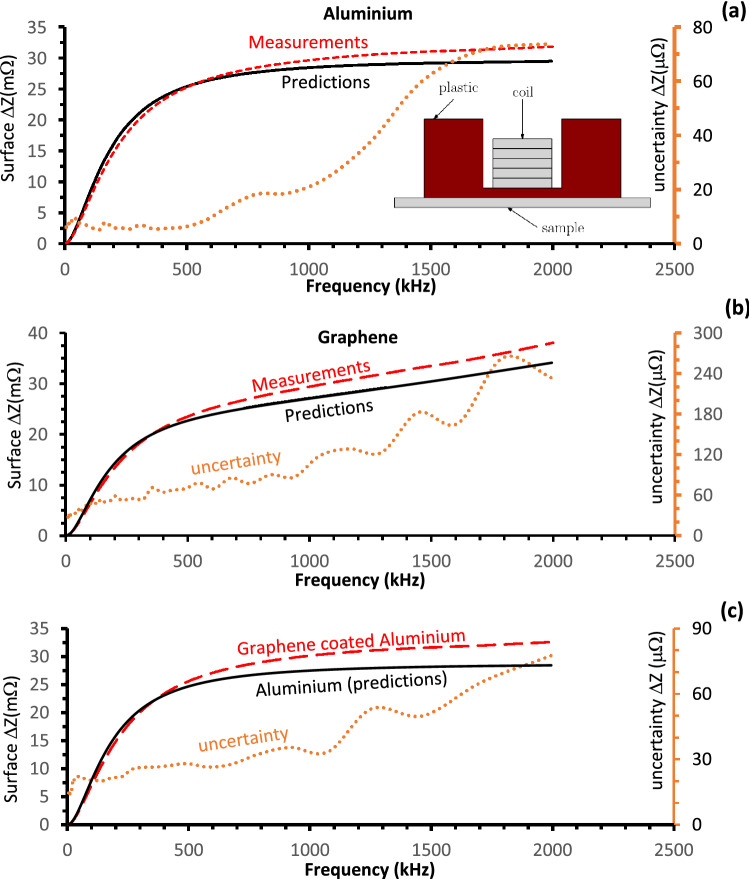
The ∆ZF measured (dashed lines) and predicted (solid lines) using expression (2). The dotted lines, in all figures, illustrate the measurement uncertainties with the scale shown on the right- hand side. The uncertainties are 2 orders of the magnitude smaller as compared with the value of measurements, increasing slightly as the frequency drops to below 50 kHz. The dependences are **(a)** aluminium; **(b)** graphene; **(c)** 3.5 nm layer of the graphene on the 16.9 μm aluminium foil. **(c)** The measurements are compared with the predictions observed for pure aluminium foil.

In Fig. 4a,b the measured (dashed-lines) impedances versus frequency for aluminium (16.31 μm thin) and graphene (28.2 μm thin) samples respectively are compared with the theoretical (solid-lines) predictions of Expression (3). The measurements have been also carried out for a two-layer composite material; specifically an aluminium foil of total 16.9 μm thickness coated with a fine 3.5 nm graphene layer (similar that shown in Fig. 1a). The results of the measurements (dashed-lines) are presented in Fig. 4c. The dependence strongly resembles the one observed for the pure aluminium (Fig. 4c, solid line). The parameters of the samples (provided by manufacturers and measured) are shown in Table 1.

**Table 1:** Foil samples thicknesses and conductivities.

Sample	Mean thickness, μm (given, μm)	Standard error (μm)	Measurement error (μm)	Conductivity measured, S/m [given]
Aluminium	16.31 (15)	0.04	0.24	(3.4 ± 0.4)10^7^ [3.7 × 10^7^]
Graphene Sheet	28.2 (25)	0.1	0.2	(1.4 ± 0.1)10^6^ [1.5 × 10^6^]
Graphene Coated (3.5 nm) Aluminium	16.9 (16)	0.1	0.2	(3.3 ± 0.3)10^7^

**Figure 5:**
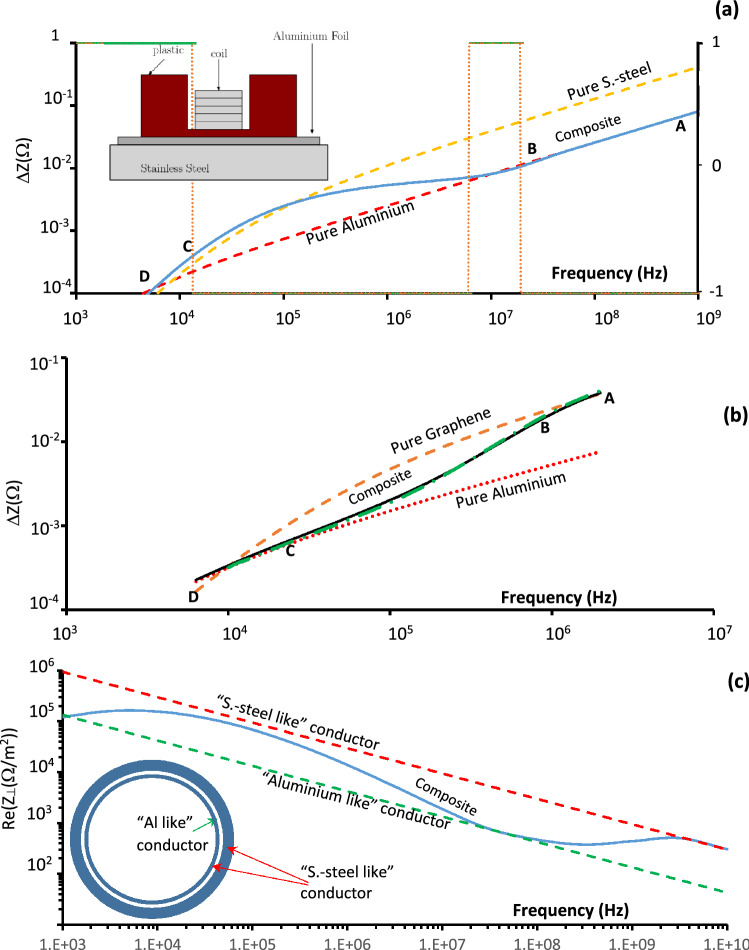
(**a**) Predicted ∆ZF dependence of solid bulk materials (dashed lines) aluminium, s.-still and composite aluminium—s.-steel material (solid line). The intervals, where the second derivative sign is changed, are shown. The inset show the schematic of experimental set up; **(b)** ∆ZF dependence measured (solid line) and predicted (dashed-dotted line) grapheme—aluminium composite material. The dashed and dotted lines show predictions for the ∆ZF dependences of the single bulk materials; **(c)** Calculated real part transverse impedance Z_⊥_ (Ω/m^2^) frequency dependences of the three-layers pipe structure. The inset shows solid grey colour cylinders which represent the “S.-steel like” high resistive layers of the conductor while white colour cylinder indicate layer of low resistive “Aluminium like” conductor).

To investigate the behaviour shown in Fig. 4c, other dual-layer materials have been studied. Figure 5a,b show the impedances of the composite materials made of: pure aluminium and stainless steel (Fig. 5a, calculated), and graphene and aluminium (Fig. 5b, measured). The inset to Fig. 5a illustrates the experimental set up used to carry out the measurements. The figures compare the impedances of pure and composite (made from these pure materials) materials observed using: theoretical approach (Fig. 5a) and experimental measurements (Fig. 5b). In Fig. 5a the predictions of the *impedance-frequency* (∆ZF) characteristics for the bulk conductors: aluminium (dotted line) and stainless steel (dashed line) are shown with the ∆ZF-characteristics (solid line) for a layer of aluminium on a semi-infinite stainless steel base (solid line). The solid line has been observed experimentally with a 51.6 μm thin pure aluminium foil laid on a 3 mm stainless-steel (s-steel) plate. Three distinguishable sections (in Fig. 5a) can be identified on the graph. In the first section (part-AB, above 16 MHz) at high frequencies ∆ZF coincides with that of a thick aluminium base. In the following, intermediate frequency BC-region (between 16 MHz and 13 kHz) ∆ZF branches out from the aluminium line and crosses the line for stainless steel at around 0.1 MHz. In the lowest frequency region, CD (below 13 kHz), the presence of the aluminium foil becomes less relevant with experimental data following the theoretical prediction for s-steel. These results explain the observations shown in Fig. 4c where a thin layer of the graphene (3.5 nm) was laid on an aluminium foil and for the range of the frequencies (0.01–2 MHz) the ∆ZF-characteristics of the foil was only measured. The graphene layer is effectively transparent as the skin depth over the full frequency range is significantly larger than the thickness of 3.5 nm. As a result Fig. 4c shows only the part of ∆ZF which corresponds to the part-CD shown in Fig. 5a.

The experimental data shown in Fig. 5b illustrates measurements of ∆ZF dependence of a 432 ± 3 µm thick layer of graphene (stack of 15 graphene sheets) on the block of the aluminium. The conductivity of the tested graphene samples (measured up to 2 MHz only) was an order of magnitude lower than that of the aluminium alloy base (similar to inset in Fig. 5a) and at higher frequencies (above 1.45 MHz) ∆ZF follows the pure graphene line as discussed above. Decreasing the frequency of the EM signal results in the ∆ZF-characteristics diverging from the graphene line and at around 10 kHz it follows the characteristics for the semi-infinite aluminium alloy block. In the figure both theoretical (solid line) and experimental (cross-line) curves are shown and the convergence between theoretical predictions and the experimental data is clear.

One of the observed features of the ∆ZF-characteristics is the change of the curvature (change of the second derivative’s sign) and the frequency positions of the points where curvature changes can be found:

3 $$ \frac{{\partial^{2} }}{{\partial \omega^{2} }}\Delta Z\left( {\omega ,\sigma ,l_{i} } \right) = 0 $$

For a single bulk conductor in the frequency range considered, the second derivative is normally negative (as observed in the range of the frequencies considered). It was measured (Fig. 5b)that only when a conductor becomes partially transparent the sign of the ∆ZF second derivative changes (point-B, 1.6 MHz). The points where the sign changes are indicated in Fig. 5a by dotted lines and the interval with positive (+ 1) and negative (− 1) values are shown using the right-hand side axis. For a “transient” section (part-BC, Fig. 5a) the sign of the second derivative may vary (at 5.2 MHz); the detailed behaviour of ∆ZF in this region is outside the scope of the paper and will be investigated in the following works. The sign changes from negative to positive (point B) as soon as the first foil becomes “partially transparent” and it changes again when it becomes “fully transparent” (point C, 13 kHz) and only the properties of the following layers become important. Condition (3) can be used to define the boundaries of full transparency of the foil (part-DC); partial transparency (part-CB) and full surface reflection (part-BA). These definitions can be useful when designing and studying composite materials, made from different foils or metamaterials, providing guidance with selecting foils of the required thickness and conductivity while minimising the thickness and bulk density of the designed composite material. The feature observed opens the possibility to design materials with a frequency selective conductivity (FSC) i.e. oscillating ∆ZF-characteristics.

To illustrate this a three-layer composite material with cylindrical geometry has been investigated. A schematic of the layout is shown in the inset of Fig. 5c and the chosen layers were 10 µm inner layer of a conductor with conductivity 10^6^ S/m (stainless steel); a 15 µm thick middle of a “good” conductor with conductivity 5 × 10^7^ S/m (aluminium) and the a final, semi-infinite external layer of the same conductivity (10^6^ S/m) as the first inner conductor. The pipe of such a wall composition could be used to transport relativistic charge-particle beams and the transverse beam coupling impedance has been calculated^33^. The results are shown in Fig. 5c where one can see the appearance of the “high conductivity zone” between 1 MHz and 1 GHz and “low conductivity gaps” above 1 GHz and below 10 MHz. The dimensions of the zone and gaps can be controlled, if necessary, by changing the geometrical and physical parameters of the layers which could be used to mitigate certain beam instabilities.

**Figure 6:**
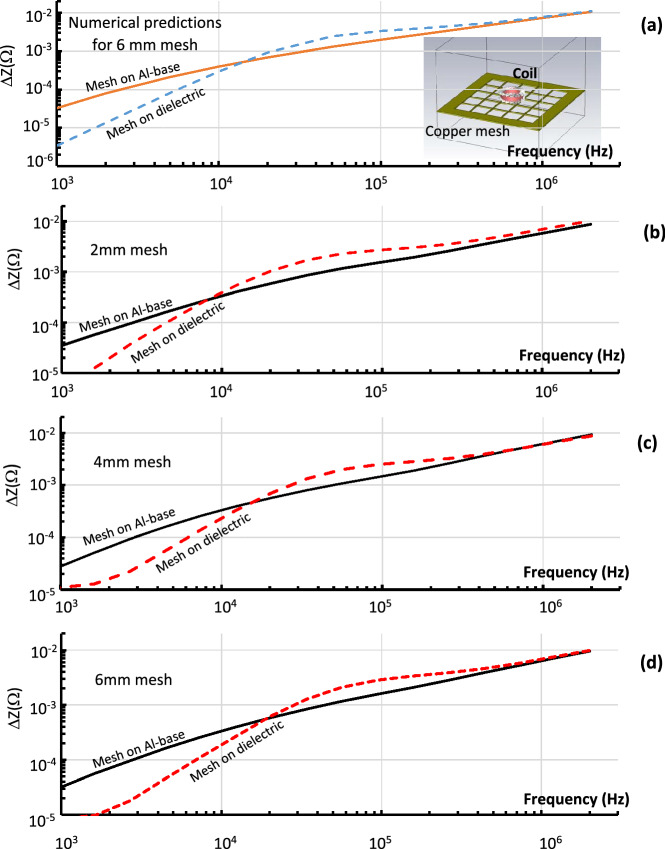
The ∆ZF dependence **(a)** simulated (δ*l* = 6 mm) and **(b**–**d)** measured for different samples of the copper mesh. The results show ∆ZF dependences if the mesh was positioned on the top of aluminium base (solid lines) and on the top of a dielectric base (dashed line). The dependences observed for meshes with **(b)** δ*l = *2 mm; **(c)** δ*l* = 4 mm; **(d)** δ*l* = 6 mm.

**Figure 7:**
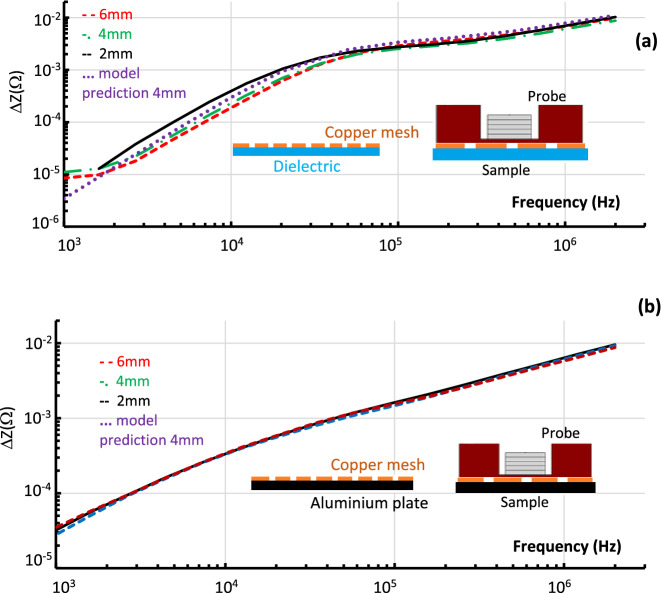
Comparison of the ∆ZF dependences (as in Fig. 6) for **(a)** isolated meshes i.e. positioned on the dielectric plate; **(b)** the meshes are positioned on the top of the an aluminium plate.

To study the application of metamaterials^1–13^ numerical and experimental studies of copper meshes^8^ (Fig. 1b and inset to Fig. 6a) have been carried out. Three samples (Fig. 1b) with the thickness 0.4 mm, ε = 0.3 mm and δ*l* = (2 mm, 4 mm, 6 mm) were investigated. Numerical studies of the copper mesh (δ*l* = 6 mm) were carried out using the low frequency solver of CST. The simulated ∆ZF-characteristics of the composite materials located on an aluminium or dielectric base, are presented by the solid and dashed lines respectively in Fig. 6a. The differences can be seen between the ∆ZF dependences at low and intermediate frequencies, while the convergence at high frequencies for all systems is evident. In Fig. 6b–d the measured ∆ZF-characteristics for copper meshes + dielectric base (dashed lines) and copper meshes + aluminium alloy base are shown. There is strong dependence of the ∆ZF on the mesh parameter (δ*l*), in the case of the mesh + dielectric (Fig. 7a) as the mesh is isolated and behaves as a pure metamaterial. This dependence became negligible as the mesh is positioned on the aluminium alloy (Fig. 7b) which effectively negates the RLC-properties of the mesh. The last observation is important for designing new EM shieling materials with reduced density and mass. For example shielding can be achieved by using a composite made of thin ~ 1 µm foil which will be facing a relativistic charge particle beam and the mesh. It allows one to maintain the EM shielding capability of a thick foil, rigidity of the mesh while significantly reducing the effective density and the mass of the material (textured foil).

## Discussion

The results presented provide understanding of the conductivity of composite materials enabling the design of materials with the frequency selective conductivity (FSC). Also a road map to design such materials has been suggested and discussed. The theoretical, numerical and experimental evaluations of ∆ZF have been demonstrated and discussed. Good convergence of the experimental and theoretical results have been observed. Full and, partial transparency as well as full surface reflection from the finite thickness composite materials have been observed and the boundaries for each phenomena were defined. By composing a material from multiple layers with appropriately chosen thicknesses and material properties, a new material with specified ∆ZF-characteristics can be constructed. The ∆ZF-characteristics of this material will be different from that of the bulk materials used to design it. One may use metamaterials like copper meshes (material with the periodic patterns) in combination with dielectric or another conductor to achieve the desired physical parameters. Surface patterns can also be used to create a “composite two-layer material”. The periodic patterning (perforation or texturing) effectively reduces the density of the material and changes its ∆ZF-characteristics. Thus a mesh in combination with dielectric or conductive foils can provide good EM shielding, mechanical strength and vacuum protection. This opens new possibilities to design components for radar, communication devices and particle accelerators helping to realise for example novel electronic devices and to construct sections of accelerator beam lines with effective control of the wakefields and thus improve control of the resistive wall instabilities observed at such facilities. The results presented will stimulate further studies to improve the design and functionality of the next generation of materials to broaden the applications. For the specific case of VELO, aluminium foils are more effective than the tested graphene foils, as they provide shielding to lower frequencies with a thinner material. Conductive meshes present an alternative to a solid foil with an improved mechanical rigidity and the potential for a smaller effective density. It is suggested that for the VELO detector to investigate possible use of a combination of the thin 5 µm foil and mesh with the foil facing toward the beam while mesh is toward the silicon detectors. Further work to optimise the design of such a mesh for VELO should be carried out.

## Methods

To carry out the measurements the Eddy current technique^37–44^ has been used. The Eddy currents are induced in the foils by an excitation coil; the experimental set-up for this technique and the idealised geometry are indicated in Fig. 3a. In the insets to Fig. 3a the schematic diagram of the Eddy current multi-turn pick-up coil (top-right corner) and photographs of the experimental set-up are shown. The number of the turns in the coil was 4 (left top corner inset), the coil inner and outer diameters were (7.18 ± 0.02) mm and (8.29 ± 0.02) mm respectfully, the coil thickness (*l*_2_-*l*_1_) = (2.33 ± 0.02) mm and plastic thickness *l*_1_ = (0.91 ± 0.01) mm. The enamelled copper wire was wound, glued to maintain a fixed geometry and embedded in a plastic housing to maintain a fixed distance between the sample and the coil; the inset of Fig. 5a shows a cross-sectional view of this. The plastic housing was oriented so that samples could be placed on-top of the coil, to avoid moving the apparatus between reference and sample measurements.

Eddy currents are induced in the foils with a circular drive coil, and the impedance to these currents measured using equipment such as a VNA or LCR meter; this impedance has a predictable dependence on the conductivity of the sample^44^. Above some frequency, the skin effect confines these currents to a thin layer of the sample material^35–37^, and the measured impedances are consistent with that of an infinitely thick sample. This ceases to be the case at low frequencies, where EM fields penetrate the sample and the measured impedance becomes dependent on structures behind the sample; these deviations can be exaggerated by positioning samples on a bed of material with significantly different conducting properties. This is demonstrated in Fig. 5a which shows the impedance versus frequency for an aluminium foil shielding a stainless steel base, and the predicted values for solid aluminium and stainless steel. If a coating is an effective EM shield this impedance will result from coating only and not the base material, while the opposite is true for an ineffective shield. To perform this technique, a reference measurement *Z*_ref_ of the coil impedance is made with no samples in the vicinity of the coil (i.e. coil’s free space impedance). A sample material is then positioned at the front of the coil resulting in change of the measured signal.

Measurements have been carried out in the frequency range of 0.01–2 MHz which is of particular interest at many accelerator facilities for the investigation of resistive wall instabilities and for the shielding of detectors. An IET 7600 + Precision LCR meter was used to drive the current and measure the coil impedance. All measurements were made in a room with active temperature control, on a dielectric optical style bench to provide mechanical rigidity and prevent movement of the connecting wires when changing samples. Reference measurements of the free-space coil impedance were made between sample measurements and averaged, to reduce the impact of small changes to atmospheric conditions.

The dimensions of the foil samples were defined using the following steps. The coil used for the measurements was approximated as an infinitely thin current ring of the radius 4.2 mm and the induced surface Eddy currents were assumed to have only radial dependence (no depth dependence). The absolute value of the current density was then used to calculate power deposition on the conductor

4 $$ P/L = \frac{2\pi }{{2\sigma }}\mathop \smallint \limits_{0}^{R} \left| {J\left( r \right)} \right|^{2} rdr $$

where *L* is the depth, *J* is the current density and in Fig. 3b the current density radial dependence is shown, assuming that the centre of the coil is positioned at zero, the dotted line indicates the position of the ideal coil. It was found that > 99% of the power should be deposited within a 2 cm radius and 4 cm × 4 cm samples were prepared. The inset to Fig. 3b shows the Eddy currents induced on the surface of surface of a square copper mesh and in a conductive plate, as calculated by a numerical CST LFS simulation. The arrows on the samples represent the current density excited by the coil. The arrows decay fast from the coils centre confirming the dependence observed from the semi-analytical analysis and presented in Fig. 3b. The results indicated that the samples of 4 cm × 4 cm should be large enough to ignore fringe effects. Foil thicknesses were obtained by measuring the thickness of a stack of foils with a digital micrometer, in the same temperature controlled room as the Eddy current measurements.

The EM properties of these materials and their electromagnetic transparency were studied using semi-analytical and numerical approaches allowing better understanding of the experimental data. Numerical simulations were carried out with the CST LF-FD magneto-quasi static solver by specifying a drive coil with a hollow cylindrical geometry. One notes that the Maxwell equations are scalable, and the concept presented i.e. frequency dependence of the ∆ZF-characteristics (though in this case has been observed using quasi-magnetostatic approach) can be extrapolated to higher frequencies. In this case the direct measurements of the sample’s reflection and transmission coefficients (instead of Eddy current technique) will be required. At higher frequencies the thickness of the layers from which such materials are composed would change from µm to nanometre or would have nanometre sized periodic features (texturing) on the surface or perforations. Moving up frequency to visible and EUV light will require amendments to the conductivity theory used in this work; however the general principle discussed in this paper should still be applicable.
